# Evidence for investing in parenting interventions aiming to improve child health: a systematic review of economic evaluations

**DOI:** 10.1007/s00787-022-01969-w

**Published:** 2022-03-19

**Authors:** Filipa Sampaio, Camilla Nystrand, Inna Feldman, Cathrine Mihalopoulos

**Affiliations:** 1 Department of Public Health and Caring Sciences, Uppsala University, BMC, Husargatan 3 (Entry A11), 751 22 Uppsala, Sweden; 2 Department of Epidemiology and Global Health, Umeå University, Umeå, Sweden; 3 School of Health and Social Development, Deakin Health Economics, Institute for Health Transformation, Deakin University, Geelong, Australia

**Keywords:** Economic evaluation, Cost-effectiveness analysis, Parenting, Child health, Prevention, treatment

## Abstract

A comprehensive review of the economic evidence on parenting interventions targeting different aspects of child health is lacking to support decision-making. The aim of this review is to provide an up to date synthesis of the available health economic evidence for parenting interventions aiming to improve child health. A systematic review was conducted with articles identified through Econlit, Medline, PsychINFO, and ERIC databases. Only full economic evaluations comparing two or more options, considering both costs and outcomes were included. We assessed the quality of the studies using the Drummond checklist. We identified 44 studies of varying quality that met inclusion criteria; 22 targeting externalizing behaviors, five targeting internalizing problems, and five targeting other mental health problems including autism and alcohol abuse. The remaining studies targeted child abuse (*n* = 5), obesity (*n* = 3), and general health (*n* = 4). Studies varied considerably and many suffered from methodological limitations, such as limited costing perspectives, challenges with outcome measurement and short-time horizons. Parenting interventions showed good value for money in particular for preventing child externalizing and internalizing behaviors. For the prevention of child abuse, some programs had the potential of being cost-saving over the longer-term. Interventions were not cost-effective for the treatment of autism and obesity. Future research should include a broader spectrum of societal costs and quality-of-life impacts on both children and their caregivers.

**Supplementary Information:**

The online version contains supplementary material available at 10.1007/s00787-022-01969-w.

## Introduction

There is a widespread acceptance that the interplay between the child and her environment, composed of many layers including family, peers, and social structures, contribute to the child’s development. Of all protective and risk factors influencing child development, a key part is the quality of parenting that children are exposed to [1]. Inadequate parenting, such as low levels of supervision and involvement, inconsistent rule setting, and punitive discipline, therefore, reinforce inappropriate or negative outcomes in children [2]. Conversely, a warm and close relationship where parents are supportive and use positive reinforcement are important protective factors [3, 4].

The use of parents as active agents in influencing an unwanted behavior or outcome in children may thus be beneficial, an idea that has been formative in the development of parenting interventions. Parenting interventions aim to improve child behavior through improving parenting strategies and parent–child relationships [5]. These are commonly used as a preventive or treatment measure and are largely effective strategies, where changes in parental behavior trickle down to reducing child problematic behavior. This is especially true for disruptive behavior problems [6] and child maltreatment [7]. Effects increase per level (universal, selective, and indicated) of prevention [8], and parenting interventions are effective when delivered face-to-face [9] as well as online [10]. Effects have also been seen across different country contexts [11].

Problems that parenting interventions aim to target, including disruptive behavior, child maltreatment, emotional problems, and obesity, are costly to individuals, families, and society [12, 13]. If coexisting with parental mental health problems, they may be even larger [14]. If problems are left unresolved, they may additionally result in long-term adverse consequences, including persistent mental health problems, socio-economic struggles, and criminality [15–17]. Simultaneously, to provide the best possible and equitable care to children and their families, resources need to be prioritized in light of competing alternatives. It is, thus, important that decisions on which parenting interventions are to be adopted are made not only based on effectiveness but also on whether the outcomes produced by such interventions are good *value-for-money*.

The literature on the economics of parenting interventions for improving child health dates back to the 1980s. Considerable research has been undertaken, looking at outcomes and/or costs of parenting interventions and their impact on patterns of resource use. Although informative, evaluations that investigate either outcomes or costs separately, only consider one of two important dimensions of economic evaluation, and cannot fully guide decision-makers in resource prioritization. Full economic evaluations are necessary, to compare two or more interventions in terms of costs *and* health outcomes [18].

Several reviews of economic evaluations of parenting interventions have previously been conducted. An earlier review compiled the economic evidence of parenting interventions that aimed to support families with children with or at risk of developing conduct disorder. The review included six studies but was cautionary to draw conclusions; as only three of these studies were full economic evaluations and measured different health outcomes and costs of different interventions, making comparisons difficult [19]. Duncan et al. [20] synthesized the economic evidence for parenting interventions aimed to improve parent–infant interaction. Ten studies were included in the review that concluded that the interventions could result in substantial savings, both in the short- and long-term. Another review evaluated the evidence for parenting interventions, in the UK, for preventing behavior problems in children, finding that the interventions had the potential to be cost-saving in the long-term [21].

Lacking in the literature is a comprehensive review including a broader range of child health outcomes, as well as an assessment of study quality. In addition, there has been a surge of economic evaluations of parenting programs in the last five years, more than doubling the amount of available evidence. The aim of the current systematic review is to provide an up-to-date synthesis and appraise the quality of the available health economic evidence for parenting interventions aiming to improve child health.

## Methods

### Search strategy

This systematic review was guided by the Preferred Reporting Items for Systematic Reviews and Meta-Analysis (PRISMA) guidelines [22]. An English-language literature search was undertaken in Medline, Econlit, ERIC, and PsychInfo for peer-reviewed literature published until June 2020. Search terms were informed by previous systematic reviews [19, 21] and included ‘child*’ AND ‘parent*’ AND ‘economic evaluation’ OR ‘cost benefit’ OR ‘cost effectiveness’ OR ‘cost utility’ OR ‘cost offset’ OR ‘cost minimization’. All results were exported to Mendeley version 1.19.4. The review was registered in PROSPERO: CRD42020206303.

Inclusion criteria for the review comprised: (1) studies that met the criteria for a full economic evaluation, considering both costs and outcomes of two or more alternatives; and (2) studies with a randomized or quasi-randomized controlled design with at least one parenting intervention arm aiming to improve child health-related outcomes, including measures of physical and mental health. A parenting intervention was defined as a manualized structured intervention, focusing on parenting skills and practices [5]. The following exclusion criteria were considered: (1) reviews, editorials and abstracts from conferences, (2) studies without access to full text; (3) studies where only parental outcomes and no child health-related outcomes were reported. To validate the search strategy and to find missing articles, reference lists of all systematic reviews identified in the initial search were checked for other relevant articles.

Three of the authors (FS, CN, and IF) screened one third of the titles and abstracts each. To consider inconsistencies between author assessments, a random sample of 20% of the abstracts was assessed by another author. Author agreement on article relevance was estimated based on inter-rater reliability, resulting in a Cohen’s kappa coefficient between 0.88 and 1.00, reflecting good agreement [23]. Inconsistencies were discussed to reach complete agreement. Thereafter, abstracts included were screened for full text inclusion based on inclusion criteria.

### Quality assessment

Three independent reviewers (FS, CN, and IF) assessed the quality of the studies included after full text screening using the Drummond [18] checklist. Disagreements were discussed among authors until consensus was reached. This checklist includes ten items, each with three potential responses, “yes”, “unclear”, and “no”, which were scored 1, 0.5, and 0, respectively. Items 7 and 8 have the additional potential response “not applicable”. When these items were deemed not applicable, they were excluded from the calculation of total score. A scoring system was used to calculate an average score across all applicable items, with each item weighted equally [24]. Total scores range between 0 and 1. Studies were classified into high (score 0.8–1.0), moderate (score 0.6–0.79), and poor quality (score ≤ 0.59).

### Data extraction and study classification

Data from the articles selected for inclusion were extracted using a template relevant to the study aim. Extracted items were summarized in a narrative format and included: author/year, setting, problems targeted, population, intervention(s), comparator, follow-up/time horizon, analysis perspective, costs included, outcomes (generic and clinical, and instrument), summary of results, and study quality. Studies were classified according to the type of evaluation performed. Evaluations included cost-effectiveness analyses (CEA) (using clinical outcome measures), cost-utility analyses (CUA) (using generic outcome measures, such as quality-adjusted life-years (QALY) or disability adjusted life years (DALY), which serve as common metrics that can be used to compare different interventions), cost–benefit analyses (CBA) (quantifying health outcomes in monetary terms), cost-minimization analyses (CMA) (when health outcomes are not significantly different, thus only costs are compared), and cost-consequence analyses (CCA) (when both costs and outcomes are described without incremental estimates being computed). CUA is the only evaluation type that allows for results to be compared against an established willingness-to-pay (WTP) threshold for a gained QALY. Although it could be argued that cost-offset analyses (comparing costs incurred with costs saved) are not full evaluations, these were also included, as the line between costs and outcomes if often ambiguous and some outcomes might be proxied by service use. Studies were also classified as to whether they were based on primary data or simulation modeling. Interventions were classified within the prevention spectrum (universal, selective, indicated) or as treatment. Universal interventions target the whole population; selective interventions target at-risk groups; and indicated interventions target high-risk groups with signs or symptoms of disorders, but who do not meet the full criteria for a diagnosis [25].

Three reviewers (FS, CN, and IF) independently extracted data, while a random sample of 20% of the articles were selected for review by another author. Disagreements were resolved through discussions.

## Results

### Search results

**Fig. 1:**
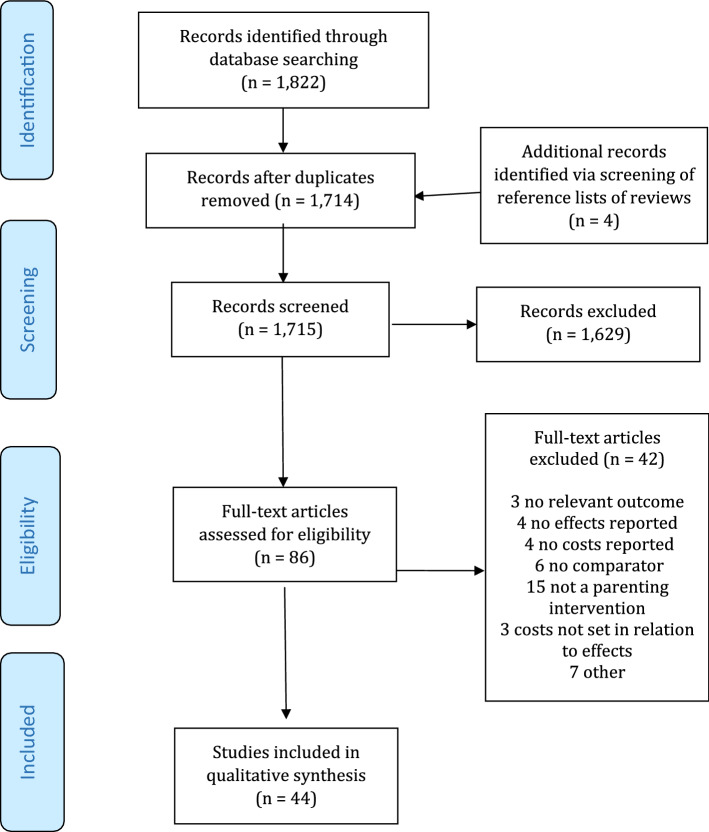
PRISMA flow diagram of study selection process

The electronic search resulted in 1714 unique publications, and four additional articles were identified when screening reference lists of relevant reviews. Figure 1 shows a flowchart of the study selection process. Based on the title and abstract review, 86 articles were selected for full text review. Of these, 42 were excluded because they did not evaluate parenting interventions (*n* = 15), did not include a comparator (*n* = 6), were not full economic evaluations and reported only costs (*n* = 4) or only effects (*n* = 4), did not have a relevant outcome (*n* = 3), did not set costs in relation to effects (*n* = 3), and were report versions of other publications or not available in full text. After the full text review, 44 studies were selected for data extraction and quality assessment.

### Quality assessment

Of the 44 studies included, a majority of the studies were rated to be of high quality (*n* = 32), 11 studies of moderate quality and 1 of poor quality. The most frequent reasons for not receiving full points among all studies was failing to include all relevant costs and outcomes (64% of studies), failing to discount costs and outcomes occurring in the future to present value (65% of studies), and failing to adequately characterize uncertainty around the cost and effect estimates (64% of studies). Additionally, moderate quality studies also often failed to pose a well-defined research question, to adequately measure and value costs and outcomes, and to address all issues of concern in the discussion (See Tables S1 and S2 in the Supplementary Appendix).

### Overview of the studies

Of the 44 included studies, most targeted child mental health (*n* = 32): 22 studies targeted externalizing behavior problems (such as attention deficit/hyperactivity disorder and conduct disorder) [26–47], five studies targeted internalizing problems (i.e., anxiety) [48–52], and five studies targeted other mental health problems including, for instance, autism, and alcohol abuse [53–57]. The remaining studies targeted child abuse (*n* = 5) [58–62], obesity (*n* = 3) [63–65], and general health (*n* = 4) [66–69]. Most interventions (*n* = 30) evaluated preventive strategies, whereas 14 studies evaluated treatment strategies. Most studies were conducted in Europe (*n* = 24) including the UK, Ireland, Sweden, and the Netherlands, followed by North America (*n* = 13) and Australia (*n* = 5). The majority of studies were CEA (*n* = 28) using clinical outcome measures, nine were CUA using QALYs or DALYs, four were CMA, three cost-offset analyses and one cost consequence analysis. Most studies were within trial evaluations (*n* = 34), a large proportion with time horizons of 1 year or less (*n* = 23). Ten studies modeled costs and benefits over longer time horizons, ranging from one year to lifetime. A variety of costing perspectives were employed, ranging from limited to fuller societal perspectives (*n* = 28) and to narrower third-party payer perspectives (*n* = 8) sometimes limited to only intervention costs. The main characteristics of the studies are summarized in Table 1, and methods and results are summarized in Table 2. All costs were converted to 2020 US$ using purchasing power parities.

**Table 1:** Characteristics of the studies included

Author and year	Setting	Problems targeted /prevention/treatment	Population	Intervention	Comparator	Follow-up
**Mental health**						
***Externalizing behavior problems***						
Nystrand et al., 2019 (1) [26]	Sweden	Externalizing behavior; indicated prevention	5–12 y.o	*Comet* (10 group sessions and 1 individual session of 2.5 h), *Cope* (10 group sessions of 2 h), *Connect* (10 sessions of 1 h), *Incredible Years* (12 sessions of 2.5 h) and *bibliotherapy* (book on parent management techniques)	Waitlist control	Until age of 18
Nystrand et al., 2019 (2) [27]	Sweden	Externalizing behavior; indicated prevention	5–12 y.o	*Comet* (10 group sessions and 1 individual session of 2.5 h), *Cope* (10 group sessions of 2 h), *Connect* (10 sessions of 1 h), *Incredible Years* (12 sessions of 2.5 h) and *bibliotherapy* (book on parent management techniques)	Waitlist control	Until age of 65
French et al., 2018 [38]	USA	Disruptive behaviors; treatment	4–6 y.o	Parent–Child Interaction Therapy (PCIT) delivered at home. Average of 18 sessions	Parent–Child Interaction Therapy (PCIT) delivered in clinic, 11 sessions	3–4 months
Gross et al., 2019 [41]	USA	Externalizing behavior; indicated and selective prevention	2–5 y.o with low income and predominately African American parents	The Chicago Parent Program (CPP)—12 two-hour weekly sessions in groups of approximately 10 parents led by two clinicians	Parent–Child Interaction Therapy (PCIT)—individual 1 h session parent–child coaching program led by 1 clinician	4 months
Tran et al., 2018 [42]	USA	ADHD; treatment	7–11 y.o children with ADHD-I	Psychosocial program Child Life and Attention Skills (CLAS)—included integrated parent, teacher, and child components (90-min parent group meetings, 30-min individual meetings with the parents and child, 90-min child group meetings, 30-min teacher consultation meetings)	1. Psychosocial program parent-focused treatment (PFT) as active treatment control, included the parent component from the CLAS program (90-min parent group meetings, 30-min individual meetings with the parents and child); 2. TAU—conventional treatment by community providers available to all participants	13 weeks (3 months)
Sampaio et al., 2018 [43]	Australia	Conduct disorder; treatment	5–9 y.o	Group and individual Triple P level 4. *Group—*4 group sessions of 2 h + 4 telephone consultations of 30 min + workbook. *Individual*—10 sessions of 1 h	Do-nothing	5–9 y.o followed up to age 18
Sonuga-Barke et al., 2018 [44]	UK	ADHD; treatment	Preschool children (2 y.o 9 months—4 y.o 6 months)	1. New Forest Parenting Program (NFPP)—12-week, 1.5 h sessions individually delivered; 2. Incredible Years (IY) parenting program—12-week 2–2.5 h group-based sessions and weekly phone calls	TAU (standard patterns of preschool ADHD care available ranging from parent training and education to very little support)	6 months
Olthuis et al., 2018 [45]	Canada	Disruptive behaviors; treatment	6–12 y.o	Strongest Families™ Parenting the Active Child—distance delivered behavioral intervention including 12 sessions delivered by written information and videos sent by mail and 30–40 min telephone coaching	TAU (services offered by their referring agency or other service provider)	22 months
Gardner et al., 2017 [46]	UK	Conduct problems; indicated and selective prevention	3–8 y.o (trial based) and 5 y.o (model based)	2 part economic evaluation—one short-term trial based and one long-term model-based evaluation. Effectiveness data based on individual level data from 5 trials. Thus, the intervention/control conditions varied slightly. In all trials, the Incredible Years parenting program was delivered. Mostly, the standard 12- to 14-week IY Basic parenting program was delivered	The control condition differs across trials, with some offering waiting list or treatment as usual and others offering minimal treatment (trial-based study). In the model-based part, data from the literature was used to assess the trajectory of a control group	6 months (trial based) and 5 y.o modeled until 30 years of age (model based)
Sayal et al., 2016 [47]	UK	ADHD; indicated prevention	4–8 y.o	Magic parenting program (parent-only arm and parent + teacher arm). *Parent-only arm*—3 group sessions of 2 h. *Parent* + *teacher arm*—parent-only arm plus an additional 1.5 h group session delivered to teachers	Do-nothing	6 months
Sampaio et al., 2016 [28]	Sweden	Conduct problems; indicated prevention	3–12 y.o	*Comet* (10 group sessions and 1 individual session of 2.5 h), *Cope* (10 group sessions of 2 h), *Connect* (10 sessions of 1 h), *Incredible Years* (12 sessions of 2.5 h) and *bibliotherapy* (book on parent management techniques)	WC	3 months
Sampaio et al., 2015 [29]	Sweden	Externalizing behavior; universal prevention	2–5 y.o	Triple P levels 2 and 3. Level 2—3 stand-alone 1.5 h-group seminars. Level 3—up to 4 individual sessions of 15–20 min	WC	18 months
O’Neill et al., 2013 [30]	Ireland	Conduct problems; indicated prevention	3–7 y.o	Incredible Years—12 to 14 group sessions of 2 h	WC	6 months
Bonin et al,. 2011 [31]	UK	Conduct disorder; indicated prevention	5 y.o	Evidence-based parenting program from literature (tested different delivery options: group only, individual only, 80% group + 20% individual	Do-nothing	Until age of 30
Sharac et al., 2011 [32]	UK	Severe behavior problems; selective prevention	3–8 y.o with adoptive parents	Home-based parenting interventions combined: 1. Cognitive behavioral approach from Webster-Stratton—10 sessions of 1 h; 2. Educational approach—10 sessions of 1 h (parents were given a training manual)	TAU (undefined services as usual)	6 months
Scott et al., 2010 [33]	UK	Antisocial behavior; selective prevention	6 y.o from deprived areas	28 sessions of 2.5 h: 12 sessions child behavioral program (Incredible Years), 10 sessions child literacy program (Spokes program) and 6 sessions revision	Help call line on how best to access regular services	1 year
Edwards et al., 2007 [34]	UK	Conduct problems; indicated prevention	3–4 y.o	Incredible Years—12 group sessions of 2 h	WC	6 months
Mihalopoulos et al., 2007 [35]	Australia	Conduct disorder; different levels of prevention	6–12 y.o	Triple P levels 1 to 5 (five levels of parenting support of differing intensity, where level 1 is a universal parent information strategy and level 5 is an enhanced behavioral family intervention program)	Do-nothing	Until age of 28
Foster et al., 2007 [40]	USA	Conduct problems; indicated prevention	3–8 y.o	Incredible years (different combinations of parent (PT), child (CT) or teacher training (TT))—number and length of sessions differed	WC	20 years of data
Muntz et al., 2004 [36]	UK	Severe behavior problems; treatment	2–10 y.o	Intensive practice-based parenting program—delivered by CAMHS staff undertaken by 2 consultant clinical child psychologists. A 5 h session with a child psychologist was added to the intensive treatment condition	TAU—standard treatment provided by CAMHS, which comprised of child psychiatrists, clinical child psychologists, specialist social workers and child therapists	4 years
Harrington et al., 2000 [37]	UK	Behavioral disorders; treatment	3–10 y.o	Community-based group therapy—each service used their routine interventions for behavioral disorder. In one of the two included districts, the videotape modeling parental group education program of Webster-Stratton was used. The other district used a program of parental education groups with parallel child groups	Hospital-based group therapy	1 year
Cunningham et al., 1995 [39]	Canada	Behavior problems; treatment	Families of kindergartners	Community-based group therapy, clinic-based individual therapy (12 weeks)	WC	6 months
***Internalizing behavior problems***						
Chatterton et al., 2019 [48]	Australia	Anxiety disorder; treatment	7–17 y.o	Stepped care—participants could receive up to three steps: Step 1 comprised a therapist assisted, low-intensity intervention (CBT via printed or CD-ROM materials). Therapists provided parents of children (< 13 years) with up to four 30-min telephone sessions. Adolescents (> 12 years old) received up to four 40-min calls from a therapist with the time divided between the adolescent and a parent. Step 2 followed the Cool Kids program, although number of sessions could be reduced based on the therapist's judgment. If required, Step 3 comprised up to 12 sessions of individual CBT. At each step, qualifications and experience of the therapist increased	Cool Kids program—manualized CBT program, 10 face-to-face, 1 h individual sessions over 12 weeks with a therapist	1 year
Creswell et al., 2017 (49)	UK	Anxiety disorder; treatment	5–12 y.o	GPD-CBT—brief guided parent-delivered-cognitive behavioral therapy. Parents receive up to 8 weekly sessions with a therapist (total 5 h); 4 of these sessions are face-to-face (45 min) and 4 are brief telephone reviews (15 min). Parents also receive a self-help book	SFBT—solution-focused brief therapy. Initial face-to-face session with parent and child to initiate treatment (60 min), 4 face-to-face sessions of SFBT with the child (four 45 min sessions), and a final session with child and parent (60 min; 5 h total)	6 months
Mihalopoulos et al., 2015 [50]	Australia	Anxiety; indicated prevention	3–5 y.o at screening	Group-based parenting intervention—up to 6 sessions of 1.5 h	Do-nothing	3 years and 11 years
Simon et al., 2013 [51]	Netherlands	Anxiety; indicated prevention	8–12 y.o	Screening + combination of parent or child-focused intervention. *Parent-focused*—3 group sessions of 90 min + 5 telephone sessions with each parental couple (15 min each). *Child-focused*—8 group sessions of 90 min	Do-nothing	2 years
Simon et al., 2012 [52]	Netherlands	Anxiety; indicated prevention	8–12 y.o	Parent-focused arm and child-focused arm. *Parent-focused*—3 group sessions of 90 min + 5 telephone sessions with each parental couple (15 min each). *Child-focused*—8 group sessions of 90 min	Do-nothing	2 years
**Other mental health problems**						
Lynch et al., 2017 [53]	USA	Internalizing symptoms, externalizing behavior; selective prevention	5 y.o foster children	Kids in Transition to School (KITS) consisting of two components; a 24-session school readiness group (2 h each session) for children and group-based 8-sessions bi-weekly (2 h/session) for parents	TAU (usual services available to children in a foster care control group, FCC)	1 year
Salloum et al., 2016 [54]	USA	Posttraumatic stress symptoms (PTSS); indicated prevention	3–7 y.o with PTSS	Stepped care trauma-focused cognitive behavioral therapy (SC-TF-CBT)—Step 1 consisted of three in-office therapist-led sessions (60 min), 11 parent–child meetings at-home over six weeks using a workbook. If the child responded to Step 1 the child proceeded to the maintenance phase for six weeks to practice the skills learned. If the child did not respond, s/he stepped up to Step 2, which consisted of nine TF-CBT sessions	Standard TF-CBT—12 (90-min) in-office therapist-led sessions, provided to the child with active parent involvement	3 months
Byford et al., 2015 [55]	UK	Autism; treatment	Preschool children	Communication focused therapy (Pre-school Autism Communication Trial—PACT) + TAU. The intervention consisted of an assessment session followed by 12 individual sessions of 2.5 h. Extra monthly booster sessions offered up to a maximum of 19 sessions including the assessment session)	TAU—locally provided services (e.g., pediatricians, speech and language therapists and other health, social care and education-based services)	13 months
Herman et al., 2015 [56]	USA	Mental health; selective prevention	Divorced mothers of 9–12 y.o	New beginnings program—Mother Program and Mother-Plus-Child Program. Mother Program—parenting-focused program including 11 group sessions and 2 individual sessions. Mother-Plus-Child Program—Mother Program plus an 11 sessions for children	Bibliotherapy—mothers and children received 3 books on children’s post-divorce adjustment	15 years
Spoth et al., 2002 [57]	USA	Alcohol-use disorders; universal prevention	12–13 y.o	Iowa Strengthening Families Program (ISFP): seven sessions including children in all sessions and incorporates parent–child interactive activities. Drug-Free Years program (PDFY): five-session, the same as ISFP but less parent–child interactive activities and does not include targeted children	Do-nothing	4 years
**Child abuse and neglect**						
Barlow et al., 2019 [58]	UK	Child abuse; selective prevention	Parents receiving treatment for a drug or alcohol problem and primary caregivers of children under 2.5 y.o	Parents under Pressure (PuP)—Intensive one-to-one program with 12 modules	TAU (established services across a range of sites including family support, family counseling, and parenting programs provided in a group format)	1 year
Peterson et al., 2018 [59]	USA	Child abuse; selective prevention	CPC – 3 y.o from low-income families; FNP—first-time births to low-income, unmarried mothers on Medicaid	Child–Parent Centers (CPC)—early education intervention in public schools, providing services for low-income families beginning at age 3 years through age 9 years	Nurse–Family Partnership (FNP)—home visitation program by registered nurses to first-time mothers from the prenatal period through the child’s second birthday	Lifetime
Dalziel et al., 2015 [60]	Australia	Child abuse; selective prevention	Methadone-maintained parents	Parents Under Pressure program (PUP)—up to 20 weeks (mean 10.5) of in-home individual sessions of 1–2 h, and a workbook	Combined TAU and Brief intervention. TAU—appointment with the prescribing doctor every three months and included access to a caseworker. Brief Intervention—two standard parenting sessions delivered in the clinic by the same pool of therapists who delivered the PUP program	6 months
McIntosh et al., 2009 [61]	UK	Health and social outcomes, abuse and neglect; selective prevention	Mothers at risk of abusing and neglecting their infants	Weekly visits from a trained home visitor for a total of 18 months, beginning up to 6 months antenatally	TAU (standard health and social services)	1 year
DePanfilis et al., 2007 [62]	USA	Child neglect; selective prevention	5–11 y.o	Home visiting intervention Family Connections (FC) intervention—for 3 months (FC3)	Family Connections (FC) intervention for 9 months (FC9)	6 months
**Obesity**						
Quattrin et al., 2017 [63]	USA	Obesity; treatment	2–5 y.o with overweight or obesity with parents with BMI ≥ 25	Family-based behavioral treatment (FBT)—16 sessions consisting of 1 90-min session, 3 60-min sessions, and 13 45-min sessions administered by the group leader. Additional in-person, 1:1 coaching lasting on average 20 min before or after each group meeting. Telephone counsel between meetings. Childcare provided in sessions	Attention-controlled information control (IC)—16 sessions consisting of 1 90-min session, 3 60-min sessions, and 13 45-min sessions administered by the group leader. Telephone counsel between meetings. Childcare provided in sessions	2 years
Robertson et al., 2017 [64]	UK	Obesity; treatment	6–11 y.o. overweight or obese	Families for Health, 10-week 2.5 h family-based community program with parallel groups for parents and children, addressing parenting, lifestyle, social and emotional development	TAU (Families were offered any usual care that was available in their area: a) One Body One Life—10 week 1.5 h sessions 45-min physical activity workshop and a 45-min healthy eating workshop; b) Change4Life advisors who offered one-to-one support for weight management; c) weight management program for children and young people comprising a two-step program, MEND and Choose It, Weight Watchers®, a referral to the school nurse for children where children would be weighed and measured and offered advice	1 year
Goldfield et al., 2001 [65]	USA	Obesity; treatment	8–12 y.o	Mixed intervention with group and individual treatment. Manual based group treatment with 13 sessions á 40 min for both parents and children, divided into separate groups. Individual therapy sessions of 15–20 min	Manual based group treatment with 13 sessions á 60 min for both parents and children, divided into separate groups	1 year
**General health**						
Knight et al., 2019 [66]	USA	Cognitive skills, social and behavioral skills; self-regulatory process and executive functions; selective prevention	4 y.o in Head start centers	PALS, a manual based parenting program with 16 to 20 one-on-one bi-weekly coaching sessions; TEEM, a 20 bi-weekly, two-hour/session, instructional coaching model for teachers to enhance instructional techniques in the classroom; Both TEEM and PALS	Control group (Head Start preschools)	3 years
Häggström et al., 2017 [67]	Sweden	General health; universal prevention	Parents during pregnancy, delivery and the child’s first 2 years of life	Salut Program—integrated within care-as-usual, comprising strengthening everyday practice of parenting, antenatal care, child healthcare, dental care and open preschools	TAU (established services including antenatal care, healthcare and dental care with, open preschools)	2 years
Ulfsdotter et al., 2015 [68]	Sweden	General health; universal prevention	General population	All Children in Focus parenting program—4 group sessions and 1 booster session (after 3 months) of 2.5 h. Parents also given materials from each session	WC	6 months
Simkiss et al., 2013 [69]	UK	General health; selective prevention	Parents with children aged 2–4 y.o from deprived areas	The Family Links Nurturing Program (FLNP), a 10-week course with weekly 2 h facilitated group sessions	WC	9 months, modeling 5 and 10-year time horizons

CBT, cognitive behavioral therapy; CAMHS, Child and Adolescent Mental Health Services; TAU, treatment as usual; WC, waitlist control, y.o, year olds

**Table 2:** Summary of methods and results of the studies included

Author, year	Evaluation framework	Analysis perspective	Costs included	Outcome (instruments)	Results (2020 $US)^c^	Quality
**Mental health**						
***Externalizing behavior problems***						
Nystrand et al., 2019 (1) [26]	CUA, model	Limited societal	Intervention costs, healthcare, education	DALY averted	All parenting interventions were cost-effective at a threshold of $16,417 per DALY in relation to the WC. COPE and bibliotherapy strongly dominated the other options. An ICER of $2877 per additional DALY for COPE, in relation to bibliotherapy	High
Nystrand et al., 2019 (2) [27]	Cost-offset, model	Limited societal	Intervention costs, health care, education, productivity losses for children	Monetary benefits	Benefit–cost ratios above unity for all interventions, with estimates between 6.48 and 17.18 per $1 invested for the four parenting programs and substantially larger benefit–cost ratio for bibliotherapy. COPE generated the largest net present values	High
French et al., 2018 [38]	CEA, quasi-experimental	Third party payer (client, provider, administrator)	Intervention costs, time and travel	Changes in ECBI-scores	Clinic PCIT cost less per one-point decrease in negative behaviors, from provider ($25.29 vs $72.60) and overall (client+provider+administrator) ($42.30 vs $83.94) perspective than PCIT at home. From the overall perspective, clinic PCIT cost only 49% of home PCIT per child that moved to normal behavior ranges ($2092 vs $4258). From the provider costing perspective, clinic PCIT cost only 29% of home PCIT per normalized child ($1056 vs $3681)	High
Gross et al., 2019 [41]	CMA, RCT	Limited societal	Intervention costs, healthcare, childcare, productivity losses for parents, travel costs	Clinically meaningful non-inferiority margin on CBCL scores	CPP was non-inferior to PCIT (similar outcomes). Costs in the CPP group were significantly lower than in the PCIT	Moderate
Tran et al., 2018 [42]	CEA, RCT	Modified societal	Intervention costs, childcare, parents' time attending meetings and helping children with homework	ADHD-I cases resolved (Parent and teacher completed CSI)	ICERs per ADHD-I cases resolved: $5461 for CLAS versus TAU, $4409 for PFT versus TAU, and $6824 for CLAS versus PFT. Streamlining the model resulted in an ICER of $40 for CLAS compared to PFT	Moderate
Sampaio et al., 2018 [43]	CUA, model	Healthcare sector	Intervention costs, time and travel for parents, health cost offset of treating a case of conduct disorder	DALY averted (ECBI)	Triple P was cost-effective at a WTP of $34795 per DALY averted, when delivered in a group format (ICER = $778 per DALY averted, probability of cost-effectiveness of 99.5%); and in an individual format (ICER = $15,744 per DALY averted, probability of cost-effectiveness 99.2%)	High
Sonuga-Barke et al., 2018 [44]	CMA, RCT	Societal	Intervention costs, healthcare, extra educational support, social services and productivity losses for parents	SNAP-IV mean scores	No differences between NFPP and IY in clinical effectiveness. Individually delivered NFPP was less costly to deliver than IY	High
Olthuis et al., 2018 [45]	CEA, RCT	Limited societal	Intervention costs, healthcare, foster care, extra educational support	0.1 standard effect size incremental improvement in CBCL scores	Strongest Families was associated with greater improvement in CBCL scores and lower costs (although not significant)	High
Gardner et al., 2017 [46]	CEA, trial based and model	Limited societal	Trial: Intervention costs, community health services (including primary care), hospital services, specialist mental health services, social care, foster care) and voluntary sector. Model: NHS, social services departments, Department for Education, voluntary sector, criminal justice system, health impacts of crime and benefits payments	Trial: Point reduction on ECBI Intensity scale. Model: case/non case determined with the ECBI Intensity scale	The IY had an 80% probability of cost-effectiveness at a WTP of $230 per 1-point improvement on the ECBI intensity scale. The long-term model showed total cost savings to society between $1590 and $13,364. With a lower cost trajectory, the intervention costs outweigh the societal savings	High
Sayal et al., 2016 [47]	CEA, CUA, RCT	UK NHS and personal social services and societal	Intervention costs, healthcare, education, social care, childcare, informal care, parents productivity losses	Point change on Conner’s ADHD Rating Scale; QALY (EQ-5D-Y, CHU9D)	ICER per point change on parent-rated Conner’s ADHD scale compared to control; NHS perspective: $48 for parent-only arm and $221 for parent + teacher arm, societal perspective: $70 for parent-only arm and $375 for parent + teacher arm. ICER per QALY (EQ-5D-3L); NHS perspective: $6527 per QALY for parent-arm only, societal perspective: $9923 per QALY for parent-arm only	High
Sampaio et al., 2016 [28]	CEA, RCT	Third party payer with parents time	Intervention costs, parents time	Recovered case of conduct problems (ECBI)	Cope, Comet, Incredible Years and bibliotherapy reduced conduct problems compared to WC, with bibliotherapy being the cheapest. Comet entailed better outcomes and higher costs than bibliotherapy, ICER = $9264 per recovered case	High
Sampaio et al., 2015 [29]	CMA, RCT	Municipality payer	Intervention costs	Point reduction in ECBI score	No significant differences between intervention and WC at follow-up	Moderate
O’Neill et al., 2013 [30]	CEA, RCT	Department of Health	Intervention costs, healthcare, special education and social services	Point reduction in ECBI score	ICER of $99 per 1-point improvement on ECBI intensity score. Estimates it would cost almost $10,818 to bring a child with the highest ECBI score to below clinical-cut off	
Bonin et al,. 2011 [31]	Cost-offset, model	Public sector and societal	Intervention costs, healthcare, social care, education, voluntary sector, crime	Monetary benefits (ECBI)	Cost savings to society over 25 years per family: $28,994	High
Sharac et al., 2011 [32]	CEA, RCT	Societal	Intervention costs, healthcare, social care, education, parents productivity losses	Point reduction in SDQ total difficulties score	No significant differences in either costs or outcomes between interventions	Moderate
Scott et al., 2010 [33]	CEA, RCT	Third party payer	Intervention costs	Standard deviation improvement (PACS)	Significant improvements in antisocial behavior, ADHD symptoms, and reduction in oppositional defiant disorder diagnosis. ICER was $8692 per standard deviation improvement	Moderate
Edwards et al., 2007 [34]	CEA, RCT	Multi-agency public sector	Intervention costs, healthcare, special education and social services	Point reduction in ECBI score	ICER of $147 per 1-point improvement on ECBI intensity score. Estimates it would cost almost $11,017 to bring a child with the highest ECBI score to below clinical-cut off	High
Mihalopoulos et al., 2007 [35]	CEA, model	Government as third-party payer	Intervention costs, costs of conduct disorder (foster and residential care in childhood, special education, state benefits in adulthood, breakdown of relationship (domestic violence and divorce), health, and crime)	Number cases averted (ECBI)	Triple P is likely to be cost-saving over the long-term if at least 7% of cases of CD were averted. Net benefits estimated at $31.3 million based on a minimum estimated reduction of 25% of cases of CD	High
Foster et al., 2007 [40]	CEA, combination of different trials	Third party payer	Intervention costs	Unit improvement in PBQ, DPICSR	At a WTP of $4168 per unit improvement in the outcome measure for problems in school, a combination of parent and teacher therapy had a probability of cost-effectiveness above 50%. At about the same WTP, for problems at home, the combination of three components: parent, teacher and child therapy had about the same probability ofcost-effectiveness	Moderate
Muntz et al., 2004 [36]	CEA, RCT	Societal	Intervention costs, healthcare, special education and social services	Point reduction on scale of CBCL	No significant differences in costs or outcomes between an intensive psychological intervention and standard treatment	High
Harrington et al., 2000 [37]	CEA, RCT	Societal	Intervention costs, healthcare, social services, education, voluntary and private sectors, childcare, travel cost	Change in ECBI scores	There were no significant differences between community and hospital-based therapy in terms of costs or outcomes	High
Cunningham et al., 1995 [39]	CCA, RCT	Third party payer with time and travel	Intervention costs, parents time and travel	Point reduction on CBCL scales	Community-based group therapy entailed similar costs but better outcomes than clinic-based individual therapy	Moderate
**Internalizing behavior problems**						
Chatterton et al., 2019 [48]	CUA, RCT	Healthcare and societal	Intervention costs, healthcare, parents time to attend intervention, and productivity losses	QALY (CHU9D)	The three-step model of stepped care provides similar outcomes at a comparable health sector cost to face-to-face psychological therapy. It was however, less costly to deliver from a societal perspective	High
Creswell et al., 2017 [49]	CUA, RCT	Societal	Intervention costs, healthcare, social care, education, other non NHS costs, lost leisure and productivity losses by parents and children	QALY (CHU9D, EQ5D-Y)	No significant differences in clinical outcomes or QALYs. GPD-CBT associated with significantly lower societal costs. Probability of cost-effectiveness 96% at a WTP between $28,694—$36,279	High
Mihalopoulos et al., 2015 [50]	CUA, model	Healthcare sector	Intervention costs, time and travel for parents, health cost offset of treating a case of anxiety and depression	DALY averted	ICER of $6144 per DALY averted. At a WTP of $34,795 per DALY averted, 99% probability of cost-effectiveness	High
Simon et al., 2013 [51]	CEA, model	Societal	Intervention costs, direct healthcare, direct non-healthcare (informal care, other help, nursery), productivity losses for parents (absence from work) and children (absence from school), loss leisure time parents and children, out of pocket (medication, transportation)	ADIS improved child	Screening and differentially offering a parent-focused intervention to children of anxious parents, or a child-focused intervention to children of non-anxious parents yielded an ICER of $119 per ADIS improved child compared to do-nothing	High
Simon et al., 2012 [52]	CEA, RCT	Societal	Intervention costs, direct healthcare, direct non-healthcare (informal care, other help, nursery), productivity losses for parents (absence from work) and children (absence from school), loss leisure time parents and children, out of pocket (medication, transportation)	ADIS improved child	The parent-focused intervention dominated the control group. The child-focused intervention had an ICER of $6220 per ADIS improved child, in comparison to the control group	High
**Other mental health problems**						
Lynch et al., 2017 [53]	CEA, RCT	Public sector	Intervention costs, healthcare, education and social services	Days free of internalizing or externalizing symptoms (CBCL)	KITS significantly increased days free from internalizing and externalizing symptoms compared to FCC, with no impact on usual services. For one internalizing problem free day, the ICER is $68 for KITS in comparison to care as usual. For an externalizing problem free day, the estimate is $67	High
Salloum et al., 2016 [54]	CEA, RCT	Societal	Intervention costs, insurance co-payments/deductibles, time and travel costs, productivity losses for parents	PTS symptom severity (Posttraumatic stress subscale of the TSCYC)	SC-TF-CBT was non-inferior and entailed significantly lower societal costs than standard TF-CBT	Poor
Byford et al., 2015 [55]	CEA, RCT	Public sector, societal	Intervention costs, healthcare, social care, education, childcare, informal care, parents productivity losses	Clinically meaningful improvement in ADOS-G	Non-significant improvements in outcome. Total cost lower when burden on parents is included. The cost and effectiveness results presented do not support the cost-effectiveness of PACT + TAU compared to TAU alone	High
Herman et al., 2015 [56]	Cost-offset, RCT	Societal	Intervention costs, healthcare, criminal justice, childcare, spiritual support, travel costs for parents	Monetary benefits	Monetary benefits per family for intervention compared to control based on one-year reductions in health/justice system costs, 15 years after the intervention, were $1336, which outweighed the cost of the intervention	High
Spoth et al., 2002 [57]	CEA, CBA, RCT	Societal	Intervention costs, societal cost of alcohol disorders: healthcare, death from car accident or violent crime, injury, incarceration, criminal activity, productivity losses	Alcohol-use disorder cases prevented (Self-report of lifetime alcohol use)	ISFP resulted in $21,224 per case of alcohol-use disorder prevented, a benefit–cost ratio of $16.35 per $1 invested, and a net benefit of $10,090 per family. For PDFY, estimates were of $34,819 per case prevented, a benefit–cost ratio of $9.97 per $1 invested, and a net benefit of $4594 per family	High
**Child abuse and neglect**						
Barlow et al., 2019 [58]	CEA, CUA, RCT	UK NHS and personal social services and societal	Intervention costs, healthcare, education, social care, legal services and other out of pocket costs borne by parents	Reduced risk of child abuse (Risk Abuse Scale from the BCAP) and QALY (EQ-5D)	Child abuse potential was significantly reduced in those receiving the PuP program. ICER per QALY: NHS perspective $52,919, societal perspective $87,337 (lower than 50% probability of cost effectiveness). ICER per unit improvement in the BCAP: NHS perspective $1558, societal perspective $2573	High
Peterson et al., 2018 [59]	CBA, model	Government payer and societal	Intervention costs, lifetime costs of maltreatment (healthcare, child welfare, special education, productivity losses, criminal justice)	Monetary benefits from reduced incidence of child abuse	Lower costs from reduced child abuse may substantially offset, but not always entirely eliminate, program implementation cost. Including victims’ lifetime lost work productivity, NFP was cost-saving from the societal perspective (benefit–cost ratio $7.18 for NFP)	High
Dalziel et al., 2015 [60]	CEA, model	Societal	Intervention costs, lifetime costs of maltreatment (healthcare, education, productivity losses, criminal justice, government expenditure on out-of-home care and protection, lost taxes, premature death and loss of quality-of-life)	Case child maltreatment avoided (CAPI)	ICER of $33,775 per case of child maltreatment avoided. Estimated net present value saving of $2.4 million for 100 families in this population treated with PuP	High
McIntosh et al., 2009 [61]	CEA, RCT	Societal	Healthcare, social services, legal costs, local authority housing costs and family out-of-pocket costs	General health, number of infants identified as maltreated and removed from the home, risk for maltreatment (Maternal sensitivity and infant cooperativeness) CARE Index)	The difference in societal costs between control and intervention arms was $6362, while for the ‘health service only’ costs the difference was $4627. Significant improvements in maternal sensitivity and infant cooperativeness, and non-significant increase in the likelihood of infants in the intervention group being removed from the home due to abuse and neglect	High
DePanfilis et al., 2007 [62]	CEA, RCT	Government payer	Intervention costs and family out-of-pocket costs connected to the intervention	Changes in CBCL score	ICER for the FC3 intervention was $495 per unit change in CBCL scores compared to $405 per unit change for the FC9 intervention	Moderate
**Obesity**						
Quattrin et al., 2017 [63]	CEA, RCT	Societal	Intervention costs, parents time and travel costs and productivity losses	Child percent over BMI (%OBMI) change	FBT for parent and child obesity shower greater significant changes in %OBMI than the IC group at larger costs. ICER for children were $130 per U %OMBI	High
Robertson et al., 2017 [64]	CUA, CEA, RCT	UK NHS and personal social services and societal	Intervention costs, healthcare, social care, educational support for children, family out-of-pocket costs, productivity losses for parents	QALY (EQ-5D-Y), change in children’s BMI *z* score	The mean ICER of Families for Health was estimated at $893,536 per QALY gained. The probability that the Families for Health program is cost-effective did not exceed 40% across a range of thresholds. The Families for Health program was dominated by TAU considering the outcome unit change in BMI *z* score	High
Goldfield et al., 2001 [65]	CMA, RCT	Third party payer	Intervention costs	Z-BMI	Mixed treatment was more expensive ($1998 vs $706) than group treatment only, whereby the group treatment cost significantly less per unit of BMI or percentage overweight change	Moderate
**General health**						
Knight et al., 2019 [66]	CEA, RCT	Third party payer	Intervention costs	SD increase in student outcomes (Average of 17 student outcomes)	ICER of $25,877 for PALS, in comparison to the control group. Both TEEM and PALS + TEAM were not effective in comparison to the control group, hence ICERs not reported	Moderate
Häggström et al., 2017 [67]	CEA, RCT	Healthcare and limited societal	Healthcare and mothers productivity losses	Avoided case with low Apgar-score (proportion of newborn with low Apgar score 5 min after delivery (< 7 points))	From both costing perspectives, the program yielded higher effects and lower costs than care-as-usual, being thus cost-saving	High
Ulfsdotter et al., 2015 [68]	CUA, RCT	Limited societal	Intervention costs, parents leisure time and time and travel	QALY (VAS (children—parent proxy), GHQ-12 parents)	The ABC program compared to the WC yield an ICER of $51,223 per QALY gained (including child and parents QALYs) and $36,817 per QALY gained including extreme utility weight values. Probability of cost-effectiveness was 50.8%	Moderate
Simkiss et al., 2013 [69]	CUA, RCT	UK NHS and personal social services and societal	Intervention costs	QALYs, changes in PedsQL—scores (SF-12-parents, PedsQL—children)	Cost per QALY gained for parents was estimated at $60,485 (range 37,221–80,694) over 5 years and $32,837 (range 20,207–43,808) over 10 years. Probability of cost-effectiveness below $36,279 was 47% at 5 years and 57% at 10 years	High

ADIS, anxiety disorder interview schedule; BCAP, brief child abuse potential inventory; BMI, body mass index; CAPI, child abuse potential inventory; CBCL, child behavior checklist; CCA, cost-consequence analysis; CEA, cost-effectiveness analysis; CHU9D, child health utility—9 dimensions; CMA, cost-minimization analysis; CUA, cost-utility analysis; DPICSR, Dyadic Parent–Child Interactive Coding System-Revised; ECBI, Eyberg Child Behavior Inventory; EQ5D-Y, Euroqol 5 dimensions youth version; ICER, incremental cost-effectiveness ratio; PACS, parent account of child symptoms; PBQ, Behar preschool behavior questionnaire; PTS, posttraumatic stress; SDQ, strengths and difficulties questionnaire; SNAP-IV, Swanson Nolan and Pelham; TSCYC, trauma symptom checklist for young children; WC, Waitlist control; WTP, willingness-to-pay

^a^QALYs estimated using Visual Analogue Scale (VAS) for children (parent proxy) and General Health Questionnaire—12 items (GHQ-12) for parents

^b^Healthcare provider with caregiver costs. Caregiver costs included the costs of foregone paid work time and out of pocket costs for transportation and child care

^c^All costs converted to 2020 US$ from original currency using a conversion rate based on Purchasing Power Parities (PPP) for gross domestic product from http://eppi.ioe.ac.uk/costconversion/default.aspx

### Evidence synthesis

#### Mental health

#### Externalizing behavior problems

Among the high-quality studies (*n* = 14), there was evidence that preventive interventions targeting children with symptoms and parenting interventions delivered as treatment, including group and individual face-to-face programs [26, 27, 31, 35, 43, 46] were cost-effective and even cost-saving for targeting externalizing behaviors. CBA analyses of some of these programs targeting prevention showed cost–benefit ratios between US$6.48 and US$17.18 per dollar invested over the long-term [27], with savings to society over a 25 year horizon of $28,994 per family [31], or $13,364 over 20 years per child [46]. Another study estimated net benefits on the population level of $31.3 million if a minimum reduction of 25% of cases of conduct problems were achieved [35]. One study found incremental cost-effectiveness ratios (ICER) of $778 per DALY averted for group therapy and $15,744 per DALY averted for individual therapy (probability of cost-effectiveness between 99.2% and 99.5%) [43]; and another study reported ICERs between $6,527 and $9,923 per QALY for a parent only intervention compared to a parent and teacher variant [47]. Well-established and disseminated parenting interventions, such as the Incredible Years [26, 27], and the Triple P—Positive Parenting Program [35, 43] were likely to be cost-effective at local WTP thresholds.

Among the moderate quality studies (*n* = 7), group-based interventions had similar outcomes at lower costs compared to individual formats [41], and interventions targeting different combinations of parent, teacher, and child formats yielded better outcomes at higher costs than care as usual [33, 40, 42, 70]. These studies were in its majority CEA using clinical outcome measures with no existing WTP values to benchmark their results against, hence no conclusions on value for money can be drawn.

#### Internalizing behavior problems

All five studies targeting internalizing behaviors were high quality. We found evidence that parenting interventions for the prevention of anxiety were cost-effective and parenting interventions delivered as treatment produced similar outcomes at lower or equal costs in relation to comparators. For example, a group-based preventive intervention, was cost-effective with an ICER of $6144 per DALY averted and 99% probability of cost-effectiveness [50]. Another study reported a probability of cost-effectiveness of 96% at a WTP between $28,694 and $36,279 for a parent-only intervention versus a parent and child intervention [49].

#### Other mental health problems

Four studies were deemed high quality. Interventions were cost-effective for the prevention of other mental health problems [56, 57] or generated better outcomes at equal costs [53]. For example, a selective intervention for divorced mothers generated long-term cost-offsets over 15 years of $1,336 per family [56]. An evaluation of two universal interventions for the prevention of alcohol abuse among youth had the potential to delay abuse onset, with benefit ratios between $9.97 and $16.35 per dollar invested [57]. No interventions were cost-effective for treating autism [55].

### Child abuse and neglect

Among the high quality studies, there was conflicting evidence about the cost-effectiveness of parenting interventions for the prevention of child abuse. For example, one study [59] found that both a home-visiting program and centers providing early education in schools and services for low-income families were likely to be cost-saving over a lifetime horizon, with a benefit–cost ratio of $7.18 per dollar invested. Another study [60] estimated a net present value saving of $2.4 million for treating 100 families in terms of cases of maltreatment prevented. Conversely, one study found the same home-visiting program not cost-effective at local WTP thresholds, with a probability of cost-effectiveness of 26.7% [58]. These two studies differed in terms of the costs included in the analysis, where the former considered a broader range of costs than the latter, including for instance productivity losses.

### Obesity

Good quality studies did not support the cost-effectiveness of parenting interventions targeting obesity or reported better outcomes at higher costs than comparators. For example, a family-based community program for parents and children, addressing parenting, lifestyle, social and emotional development was not cost-effective compared to TAU (ICER of $893,536 per QALY and 40% probability of cost-effectiveness [64]. Another study reported that a family-based behavioral treatment improved BMI and cost more than an information control [63], although cost-effectiveness cannot be inferred. A moderate quality study showed that mixed group and individual family-based treatment was cheaper than individual only treatment [65].

### General health

Evidence on the cost-effectiveness of parenting interventions targeting general health was conflicting. One good quality study reported that a population-based program integrated within care as usual targeting mothers and their children yielded higher effects and was cost-saving compared to TAU [67]. Another good quality study showed that a group-based parenting intervention was not cost-effective at local WTP thresholds (probability of cost-effectiveness of 47% at 5 years and 57% at 10 years). [69]. Two moderate quality studies did not support the cost-effectiveness of interventions [66, 68].

## Discussion

This review aimed to provide an up-to-date synthesis of the available health economic evidence for parenting interventions aiming to improve child health. In the last three decades, 44 studies on the economic value of parenting interventions, that met the inclusion criteria for this review, were published. Most of the studies targeted child mental health (*n* = 32), in particular externalizing behavior problems, followed by internalizing problems, and other mental health problems. The remaining studies targeted child abuse, obesity, and general health. Seventy percent of studies evaluated preventive interventions.

Pleasingly, most studies were of high (*n* = 32) to moderate quality (*n* = 11). Among the studies deemed high quality, parenting interventions showed good value for money, in particular for preventing child externalizing and internalizing behaviors. High-quality evaluations of widely used parenting interventions, such as the Incredible Years and the Triple P, show that they, either: (a) were cost-effective at local WTP thresholds; or, (b) could be cost-saving over the long-term. For the prevention of child abuse, some home-visiting programs had the potential of being cost-saving over a lifetime horizon. Family-based community programs targeting the treatment of obesity were not cost-effective.

Many evaluations used cost-effectiveness designs. Although informative, these studies used a variety of disease-specific outcomes that are not directly comparable for interventions targeting the same problems or interventions across different diagnostic areas. Further, the use of clinical measures undermines the likelihood of detecting improvements that may be relevant to everyday life and general wellbeing, such as improvements in quality-of-life. This is particularly important in the case of parenting interventions that may have impacts on different areas of children’s lives. Importantly, while there are established WTP threshold values for a QALY gained or a DALY averted, no such threshold value exists for disease-specific outcome measures, making it difficult to draw conclusions regarding the value-for-money of such interventions. To tackle these limitations, studies should include instruments that can capture health-related quality-of-life based on individuals’ preferences. There are a few multi-attribute utility instruments (MAUI) available in the literature, which can be used in children [71], that make it possible to estimate QALYs and, thus facilitate value-for-money estimations. Currently available instruments are, however, limited to children older than seven years of age (unless proxies are used). A reason as to why multi-dimensional outcomes were not included in most studies in this review might be that children were of younger ages. The most common MAUIs may, however, not always be appropriate in some contexts, such as mental health, as they may not fully capture the elements of health-related quality-of-life most relevant to these children. Despite its usefulness and economic credentials, QALYs per se may also fail to capture important clinical improvements. Disease-specific instruments can be more relevant in such contexts. Future research should focus on employing and developing instruments that can capture meaningful changes for different populations.

The studies also adopted narrow costing perspectives, often including intervention and medical-related costs but lacking broader societal costs, such as educational sector costs, other societal services, informal care and productivity losses for parents. This narrow approach is likely to miss important impacts across different sectors of society. This is especially true for evaluations in child health, since many conditions have impacts across different sectors of society, and may also require the delivery of care in non-medical settings, such as schools, home, and the community. For instance, antisocial behaviors are known to result in increased use of resources in different sectors of society, such as healthcare, educational and justice system services [12, 72, 73]. Childhood anxiety disorders and child abuse also yield large costs to society, including indirect costs stemming from productivity losses of parents due to absence from paid work [74, 75]. Importantly, narrow costing perspectives limit the comparability with other interventions that may differently impact the use of resources, and may lead to inappropriate decision-making. It is, however, recognized that capturing the full scope of costs that may be impacted by a parenting intervention is a difficult task, given that many are likely to occur as the children get older. Additionally, other factors may pose difficulties to adopting a broader costing perspective, including the lack of routine data sources available for estimations of resource use beyond health care, as well as the financial burden of added data collection and the added burden of data collection on participants. With such difficulties in mind, the latest recommendations of the second panel on cost-effectiveness in health and medicine are that both a health care and if possible a broader societal presented are presented as reference cases [76].

Another important issue when evaluating parenting interventions are spillover effects, i.e., the impacts of the interventions not only on children themselves, but also on those who can be directly affected by changes in children’s health and wellbeing—such as parents and siblings. Current guidelines from the U.S. [76], Canada [77], the UK [78], and the Netherlands [79] recommend the inclusion of family costs and “spillover effects” in economic evaluation when relevant. Including spillover effects in economic evaluation can change the value of an intervention [80]. In a review of pediatric economic evaluations, the inclusion of spillover effects contributed to the cost-effectiveness of interventions being more favorable 75% of the time [81]. In the current review, more than half of the studies included at least one type of family spillover costs (i.e., time costs or out of pocket costs) but only one included parent health outcome spillovers in the ICER estimate.

Existing economic evaluations of RCTs have quite limited time horizons, often below one year. Time horizon can strongly impact the results of an economic evaluation. On average, extending the time horizon of economic evaluations leads to more favorable estimates of value [82], which is important when the impacts of an intervention may extend into the future, as is the case for interventions in child health. Modeling studies can help address some of the issues of RCTs, through longer-term projections of estimated costs and outcomes, but should always use available evidence from real world data and assumptions.

Finally, it is important to stress the importance of planning for an economic evaluation upon study design to capture all important costs and outcomes, and use appropriate instruments to measure QALYs. This is not always the case, as a few evaluations included in this review appear to have been conducted on an ad hoc basis and lack inclusion of appropriate instruments to measure health outcomes and resource use.

## Conclusions

The existing evidence suggest that parenting interventions are likely to be a cost-effective use of societal resources, with respect to preventing child externalizing and internalizing behaviors, as well as home-visiting programs to prevent child abuse and neglect. Family-based community programs targeting the treatment of obesity were not cost-effective. Future studies should aim to capture the full health and economic impacts of child health interventions. Investment in parenting interventions is value-for-money and worth serious consideration by decision-makers.

## Author contributors

All authors contributed to the conception of the study, data collection, interpretation of results and approved the final manuscript.

### Supplementary Information

Below is the link to the electronic supplementary material.

Supplementary file1 (DOCX 46 kb)
