# Supplementary material for: Evidence for investing in parenting interventions aiming to improve child health: a systematic review of economic evaluations
Source: Eur Child Adolesc Psychiatry. 2022 Mar 19;33(2):323–55. doi: 10.1007/s00787-022-01969-w (PMC10869412; doi:10.1007/s00787-022-01969-w)
Supplement: Supplementary file 1 — Supplementary file1 (DOCX 46 kb) [file 787_2022_1969_MOESM1_ESM.docx]

**Supplementary Appendix 1**

**Table S1.** Quality assessment scores of the included papers using the Drummond checklist.

|  | **Mental Health**  Externalising behaviour problems | | | | | | | | | |
| --- | --- | --- | --- | --- | --- | --- | --- | --- | --- | --- |
| **Criteria/author** | Nystrand (2019) | Nystrand (2019) | French (2018) | Gross (2018) | Tran (2018) | Sampaio (2018) | Sonuga-Barke (2018) | Olthuis (2018) | Gardner (2017) | Sayal (2016) |
| 1. Was a well-defined question posed in answerable form? | ✓ | ✓ | ✓ | ✓ | ✓ | ✓ | ✓ | ✓ | ✓ | ✓ |
| 2. Was a comprehensive description of the competing alternatives given? | ✓ | ✓ | ✓ | ✓ | ✓ | ✓ | ✓ | ✓ | ✓ | ✓ |
| 3. Was the effectiveness of the programmes or services established? | ✓ | ✓ | ✓ | ✓ | ✓ | ✓ | ✓ | ✓ | ✓ | ✓ |
| 4. Were all the important and relevant costs and consequences for each alternative identified? | ✓ | ✓ | unclear | ✓ | ○ | ✓ | ✓ | ✓ | ✓ | ✓ |
| 5. Were costs and consequences measured accurately in appropriate physical units prior to valuation? | ✓ | ✓ | ✓ | ○ | ✓ | ✓ | unclear | ✓ | ✓ | ✓ |
| 6. Were the cost and consequences valued credibly? | ✓ | ✓ | unclear | unclear | ✓ | ✓ | unclear | ✓ | ✓ | ✓ |
| 7. Were costs and consequences adjusted for differential timing? | unclear | unclear | ○ | n.a. | ○ | ✓ | n.a. | ✓ | ✓ | n.a. |
| 8. Was an incremental analysis of costs and consequences of alternatives performed? | ✓ | ✓ | ✓ | ✓ | ✓ | ✓ | ✓ | ✓ | ✓ | ✓ |
| 9. Was uncertainty in the estimates of costs and consequences adequately characterized? | ✓ | ✓ | ✓ | ○ | ○ | ✓ | unclear | ✓ | ✓ | ✓ |
| 10. Did the presentation and discussion of study results include all issues of concern to users? | ✓ | ✓ | ✓ | unclear | ✓ | ✓ | ✓ | ✓ | ✓ | ✓ |
| **Total points** | 0.95 | 0.95 | 0.80 | 0.67 | 0.70 | 1.00 | 0.83 | 1.00 | 1.00 | 1.00 |

|  | **Mental Health**  Externalising behaviour problems | | | | | | | | | |
| --- | --- | --- | --- | --- | --- | --- | --- | --- | --- | --- |
| **Criteria/author** | Sampaio (2016) | Sampaio (2015) | O’Neill (2013) | Bonin (2011) | Sharac (2011) | Scott (2010) | Edwards (2007) | Mihalopoulos (2007) | Foster (2007) | Muntz (2004) |
| 1. Was a well-defined question posed in answerable form? | ✓ | ✓ | unclear | ✓ | unclear | unclear | ✓ | ✓ | unclear | ✓ |
| 2. Was a comprehensive description of the competing alternatives given? | ✓ | ✓ | ✓ | ✓ | ✓ | ✓ | ✓ | ✓ | ✓ | ✓ |
| 3. Was the effectiveness of the programmes or services established? | ✓ | ✓ | ✓ | ✓ | ✓ | ✓ | ✓ | ○ | ✓ | ✓ |
| 4. Were all the important and relevant costs and consequences for each alternative identified? | ○ | ○ | ✓ | ○ | unclear | ✓ | ✓ | ✓ | unclear | ✓ |
| 5. Were costs and consequences measured accurately in appropriate physical units prior to valuation? | ✓ | ✓ | ✓ | ✓ | ✓ | unclear | ✓ | ✓ | ✓ | ✓ |
| 6. Were the cost and consequences valued credibly? | ✓ | ✓ | ✓ | ✓ | ✓ | unclear | ✓ | unclear | ✓ | ✓ |
| 7. Were costs and consequences adjusted for differential timing? | n.a. | ○ | n.a. | ✓ | n.a. | n.a. | n.a. | ✓ | ○ | ✓ |
| 8. Was an incremental analysis of costs and consequences of alternatives performed? | ✓ | ✓ | ✓ | ✓ | ✓ | ✓ | ✓ | ✓ | ✓ | ✓ |
| 9. Was uncertainty in the estimates of costs and consequences adequately characterized? | ✓ | ○ | unclear | ✓ | ○ | ○ | ✓ | ✓ | unclear | ○ |
| 10. Did the presentation and discussion of study results include all issues of concern to users? | ✓ | ○ | ✓ | ✓ | ○ | ✓ | unclear | ✓ | unclear | ○ |
| **Total points** | 0.89 | 0.60 | 0.89 | 0.90 | 0.67 | 0.72 | 0.94 | 0.85 | 0.70 | 0.80 |

|  | **Mental Health**  Externalising behaviour problems | | **Mental health** Internalising behaviour problems | | | | | **Mental health** Other mental health problems | | |
| --- | --- | --- | --- | --- | --- | --- | --- | --- | --- | --- |
| **Criteria/author** | Harrington (2000) | Cunningham (1995) | Chatterton (2019) | Creswell (2017) | Mihalopoulos (2015) | Simon (2013) | Simon (2012) | Lynch  (2017) | Salloum  (2016) | Byford  (2015) |
| 1. Was a well-defined question posed in answerable form? | ✓ | ✓ | ✓ | ✓ | ✓ | ✓ | ✓ | ✓ | ✓ | ✓ |
| 2. Was a comprehensive description of the competing alternatives given? | ✓ | ✓ | ✓ | ✓ | ✓ | ✓ | ✓ | ✓ | ✓ | ✓ |
| 3. Was the effectiveness of the programmes or services established? | ✓ | ✓ | ✓ | ✓ | ✓ | ✓ | ✓ | ✓ | ✓ | ✓ |
| 4. Were all the important and relevant costs and consequences for each alternative identified? | ○ | ✓ | ✓ | ✓ | ✓ | ✓ | ✓ | ✓ | ○ | ✓ |
| 5. Were costs and consequences measured accurately in appropriate physical units prior to valuation? | ✓ | ○ | ✓ | ✓ | ✓ | ✓ | ✓ | ✓ | ○ | ✓ |
| 6. Were the cost and consequences valued credibly? | ✓ | unclear | ✓ | ✓ | ✓ | ✓ | ✓ | ✓ | unclear | ✓ |
| 7. Were costs and consequences adjusted for differential timing? | ✓ | n.a. | ✓ | n.a. | ✓ | ✓ | ✓ | n.a. | n.a. | n.a. |
| 8. Was an incremental analysis of costs and consequences of alternatives performed? | ✓ | ✓ | ✓ | ✓ | ✓ | ✓ | ✓ | ✓ | ✓ | ✓ |
| 9. Was uncertainty in the estimates of costs and consequences adequately characterized? | unclear | ○ | ✓ | ○ | ✓ | ✓ | ✓ | ✓ | ○ | ✓ |
| 10. Did the presentation and discussion of study results include all issues of concern to users? | ✓ | ✓ | ✓ | ✓ | ✓ | unclear | ✓ | ✓ | unclear | ✓ |
| **Total points** | 0.85 | 0.72 | 1.00 | 0.89 | 1.00 | 0.95 | 1.00 | 1.00 | 0.56 | 1.00 |

|  | **Mental Health** Other mental health problems | | **General Health**  Child abuse and neglect | | | | | **General Health** Obesity | | |
| --- | --- | --- | --- | --- | --- | --- | --- | --- | --- | --- |
| **Criteria/author** | Herman  (2015) | Spoth  (2002) | Barlow (2019) | Peterson (2018) | Dalziel (2015) | McIntosh (2009) | DePanfilis  (2007) | Quattrin (2017) | Robertson (2017) | Goldfield (2001) |
| 1. Was a well-defined question posed in answerable form? | ✓ | ✓ | ✓ | ✓ | ✓ | ✓ | ✓ | ✓ | ✓ | unclear |
| 2. Was a comprehensive description of the competing alternatives given? | ✓ | ✓ | ✓ | ✓ | ✓ | ✓ | ✓ | ✓ | ✓ | ✓ |
| 3. Was the effectiveness of the programmes or services established? | ✓ | ✓ | ✓ | unclear | ✓ | ✓ | ✓ | ✓ | ✓ | ✓ |
| 4. Were all the important and relevant costs and consequences for each alternative identified? | unclear | ✓ | ✓ | ✓ | ✓ | ✓ | ○ | ○ | ✓ | ○ |
| 5. Were costs and consequences measured accurately in appropriate physical units prior to valuation? | ✓ | unclear | ✓ | ✓ | ✓ | ✓ | ✓ | ✓ | ✓ | ✓ |
| 6. Were the cost and consequences valued credibly? | ✓ | unclear | ✓ | ✓ | ✓ | ✓ | ✓ | ✓ | ✓ | unclear |
| 7. Were costs and consequences adjusted for differential timing? | ✓ | ✓ | n.a. | ✓ | n.a. | ✓ | n.a. | unclear | ✓ | n.a. |
| 8. Was an incremental analysis of costs and consequences of alternatives performed? | ✓ | ✓ | ✓ | ✓ | ✓ | ✓ | ✓ | ✓ | ✓ | ○ |
| 9. Was uncertainty in the estimates of costs and consequences adequately characterized? | ✓ | ✓ | ✓ | ✓ | ✓ | ✓ | ○ | unclear | ✓ | ✓ |
| 10. Did the presentation and discussion of study results include all issues of concern to users? | ✓ | unclear | ✓ | ✓ | ✓ | ✓ | ✓ | ✓ | ✓ | unclear |
| **Total points** | 0.95 | 0.85 | 1.00 | 0.95 | 1.00 | 1.00 | 0.78 | 0.80 | 1.00 | 0.61 |

|  | **General Health**  General health | | | |
| --- | --- | --- | --- | --- |
| **Criteria/author** | Knight (2019) | Häggström (2017) | Ulfsdotter (2015) | Simkiss (2013) |
| 1. Was a well-defined question posed in answerable form? | ✓ | ✓ | unclear | ✓ |
| 2. Was a comprehensive description of the competing alternatives given? | ✓ | ✓ | ✓ | ✓ |
| 3. Was the effectiveness of the programmes or services established? | ✓ | unclear | ✓ | ✓ |
| 4. Were all the important and relevant costs and consequences for each alternative identified?* | unclear | ○ | ○ | ✓ |
| 5. Were costs and consequences measured accurately in appropriate physical units prior to valuation? | ✓ | ✓ | unclear | unclear |
| 6. Were the cost and consequences valued credibly? | ✓ | ✓ | unclear | ✓ |
| 7. Were costs and consequences adjusted for differential timing? | unclear | ✓ | n.a. | ○ |
| 8. Was an incremental analysis of costs and consequences of alternatives performed? | ○ | ✓ | ✓ | ✓ |
| 9. Was uncertainty in the estimates of costs and consequences adequately characterized? | ✓ | ✓ | unclear | ✓ |
| 10. Did the presentation and discussion of study results include all issues of concern to users? | unclear | ✓ | unclear | ✓ |
| **Total points** | 0.78 | 0.85 | 0.61 | 0.85 |

*Costs were deemed relevant as those related to the costing perspective chosen; consequences were deemed relevant as those related to the problem aimed at preventing by the intervention. Cost-effectiveness analyses received full points had they included a clinical outcome; cost-utility analyses had they included a generic outcome such as QALY or DALY; cost-benefit analysis had they included monetary outcomes.

**Table S2.** Study characteristics and amount of studies that reported them.

| **Study characteristics** | **Inclusion (%)** |
| --- | --- |
| 1. Was a well-defined question posed in answerable form? | 86% |
| 2. Was a comprehensive description of the competing alternatives given? | 100% |
| 3. Was the effectiveness of the programmes or services established? | 93% |
| 4. Were all the important and relevant costs and consequences for each alternative identified?* | 64% |
| 5. Were costs and consequences measured accurately in appropriate physical units prior to valuation? | 82% |
| 6. Were the cost and consequences valued credibly? | 77% |
| 7. Were costs and consequences adjusted for differential timing? | 65% |
| 8. Was an incremental analysis of costs and consequences of alternatives performed? | 95% |
| 9. Was uncertainty in the estimates of costs and consequences adequately characterized? | 64% |
| 10. Did the presentation and discussion of study results include all issues of concern to users? | 73% |

*Costs were deemed relevant as those related to the costing perspective chosen; consequences were deemed relevant as those related to the problem aimed at preventing by the intervention. Cost-effectiveness analyses received full points had they included a clinical outcome; cost-utility analyses had they included a generic outcome such as QALY or DALY; cost-benefit analysis had they included monetary outcomes.
